# Molecular features of the complementarity determining region 3 motif of the T cell population and subsets in the blood of patients with chronic severe hepatitis B

**DOI:** 10.1186/1479-5876-9-210

**Published:** 2011-12-08

**Authors:** Jiezuan Yang, Jianqin He, Haifeng Lu, Li Wei, Sujun Li, Baohong Wang, Hongyan Diao, Lanjuan Li

**Affiliations:** 1 State Key Laboratory for Diagnosis and Treatment of Infectious Diseases, First Affiliated Hospital, College of Medicine, Zhejiang University, Hangzhou 310003, China; 2 Department of Geriatric, First Affiliated Hospital, College of Medicine, Zhejiang University, Hangzhou 310003, China

## Abstract

**Background:**

T cell receptor (TCR) reflects the status and function of T cells. We previously developed a gene melting spectral pattern (GMSP) assay, which rapidly detects clonal expansion of the T cell receptor β variable gene (TCRBV) in patients with HBV by using quantitative real-time reverse transcription PCR (qRT-PCR) with DNA melting curve analysis. However, the molecular profiles of TCRBV in peripheral blood mononuclear cells (PBMCs) and CD8^+^, CD8^- ^cell subsets from chronic severe hepatitis B (CSHB) patients have not been well described.

**Methods:**

Human PBMCs were separated and sorted into CD8^+ ^and CD8^- ^cell subsets using density gradient centrifugation and magnetic activated cell sorting (MACS). The molecular features of the TCRBV CDR3 motif were determined using GMSP analysis; the TCRBV families were cloned and sequenced when the GMSP profile showed a single-peak, indicative of a monoclonal population.

**Results:**

The number of skewed TCRBV in the CD8^+ ^cell subset was significantly higher than that of the CD8^- ^cell subset as assessed by GMSP analysis. The TCRBV11 and BV7 were expressed more frequently than other members of TCRBV family in PBMCs and CD8^+^, CD8^- ^subsets. Also the relatively conserved amino acid motifs were detected in the TCRBV22, BV18 and BV11 CDR3 in PBMCs among patients with CSHB.

**Conclusions:**

The molecular features of the TCRBV CDR3 were markedly different among PBMCs and CD8^+^, CD8^- ^cell subsets derived from CSHB patients. Analysis of the TCRBV expression in the CD8^+ ^subset was more accurate in assessing the status and function of circulating T cells. The expression of TCRBV11, BV7 and the relatively conserved CDR3 amino acid motifs could also help to predict and treat patients with CSHB.

## Background

Hepatitis B virus (HBV) infection remains a global health problem with more than 350 million chronically infected people worldwide; approximately 1 million people die from HBV-related diseases every year [1]. Notably, chronic severe hepatitis B (CSHB) is associated with a high mortality rate; however, its pathogenesis is not well understood. Although antiviral treatment can suppress viral replication, it does not promote complete clearance of HBV. Therefore, clarifying the pathogenesis of hepatitis B is particularly important. HBV-related pathology is mainly mediated by the immune response to infection [2,3]. HBV-specific cytotoxic T cell (CTL) recognition and killing of infected hepatocytes is believed to be a key factor in infection-associated liver damage.

T cell responses can be directed towards a variety of specific antigens due to the diversity of the T cell receptor (TCR) repertoire. T cells of different specificity express different complementarity determining region 3 (CDR3) that vary in length or sequence [4,5]. Measuring the frequency of specific CDR3 sequences can reflect the degree of T cell clonal expansion and provide some understanding of T cell function.

In recent years, exploring TCR expression during chronic viral infections has become a hot topic in the field of infectious disease. Furthermore, this is the basis of applied research to characterize TCR profiles in a variety of diseases and of studies that have shown that T cells with transformed genes can be used to treat advanced leukemia, a strategy that has achieved results in the clinic [6]. In this study, lymphocyte subsets were sorted using magnetic bead separation technology, and the distribution features of the T cell receptor β variable gene (TCRBV) CDR3 in PBMCs from patients with CSHB were investigated. Additionally, the CDR3 motif expressed in CD8^+ ^and CD8^- ^cell subsets was also characterized.

## Materials and methods

### Enrollment of patient population

Forty-two patients with CSHB admitted to the Department of Infectious Disease, First Affiliated Hospital, College of Medicine, Zhejiang University, between September 2010 and March 2011, were included in the present study. All subjects had been HBsAg-positive for at least 12 months. Co-infection with human immunodeficiency virus (HIV), hepatitis A virus (HAV), hepatitis C virus (HCV), hepatitis D virus (HDV), and hepatitis E virus (HEV) were excluded by laboratory testing. The patients with CSHB had a serum alanine aminotransferase (ALT) level > 40 IU/L, a total bilirubin (TBiL) level > 170 μmol/L, and a plasma prothrombin activity (PTA) < 40% [7]. Patient characteristics at the time of the study are shown in Table 1. Peripheral blood samples were collected after informed consent was obtained from each patient and healthy control. This study was conducted according to the guidelines of the Declaration of Helsinki; the Zhejiang University medical ethics committee approved all procedures involving human subjects.

**Table 1:** Clinical features of the CSHB patients at entry to the study.

Characteristics	All patients (n = 42)
Sex (male/female)	29/13
Mean age (in years)†	45.6 ± 8.3
Duration of infection (years)†	14.2 ± 9.1
ALT level (IU/L)†	68.8 ± 75.3
Total bilirubin level (μmol/L)†	363.2 ± 173.5
HBV DNA (lg, copies/mL)†	4.5 ± 1.6
HBeAg-positive patients‡	16 (38.1%)
HBV genotypes	15B, 27C

CSHB, chronic severe hepatitis B; ALT: alanine amino transferase; Normal values: ALT, ≤ 40 IU/L; total bilirubin, ≤ 21 μmol/L. †Data are expressed as mean ± SD; ‡ Data are expressed as no. (%).

### Study design

GMSP analyses were performed using PBMCs from 27 patients with CSHB, and PBMCs from 15 patients were sorted into CD8^+ ^cells and CD8^- ^cells (i.e., the remaining cell fraction) using immunomagnetic beads, then the TCRBV profile was assessed. The skewed rates of each biased TCRBV families were calculated, and the monoclonal TCRBV was cloned and sequenced. Additionally, the relationship among the skewed TCRBV families of PBMCs and CD8^+^, CD8^- ^cell subsets was analyzed.

### Biochemical and serological markers evaluation

Liver function was assessed by serum ALT, AST, and TBiL levels. These assays were determined using an automatic biochemical analyzer (HITACHI 7600, Japan). The qualitative determination of HBV markers (HBsAg/anti-HBs, HBeAg/anti-Hbe and anti-HBc) and other biochemical and serological markers was performed in the Central Clinical Laboratory of our unit.

### Quantification and determining genotype of HBV DNA

The serum HBV DNA level was quantified using a real-time fluorescence quantitative commercial kit (Shenzhen PG Biotech, Shenzhen, China) with a lowest limit of detection equal to 500 viral genome copies/mL, and the HBV genotypes were determined as reported in an earlier study [8].

### PBMCs separated, CD8^+ ^cell sorted and purity assessment

Peripheral venous blood samples were obtained after liver disease was diagnosed, prior to anti-viral treatment. PBMCs from 42 CSHB patients were isolated from fresh EDTAK_2 _anti-coagulated blood using Ficoll-Paque (CEDARLANE, Netherlands) density gradient centrifugation. For 15 samples, CD8^+ ^cells were positively selected from PBMCs using anti-CD8 antibody-coated magnetic beads according to the directions of the manufacturer (Dynal Biotech, Norway), and the remaining fraction was depleted of CD8^+ ^cells (CD8^- ^cells). Purity of the separated subsets were tested by FCM analysis using PE- labeled anti- CD8 and FITC- labeled anti- CD4 monoclonal antibodies, and the CD8^+ ^cell population was found to be > 95% pure (results not shown).

### cDNA synthesis and real-time PCR

Total RNA was extracted from PBMCs (CD8^+ ^or CD8^- ^cell subsets) using a SV Total RNA Isolation System (Promega, USA) according to the manufacturer's directions. RNA purity and concentration were determined by the optical density (OD) assessed using a spectrophotometer (Bio-rad, USA). Total RNA was immediately reverse transcribed to cDNA using the RevertAid™ First Strand cDNA Synthesis Kit (MBI, EU). Briefly, 1~5 μg total RNA was reverse transcribed with OligodT18 as primer in a 20 μL reaction volume. cDNA was diluted 1:2 before being used as a template for real-time PCR with SYBR green dye. The GoTaq^® ^qPCR Master Mix (Promega, USA) was selected as the PCR kit, and the real-time PCR (dye method) reaction parameters were as follows: 2 min of incubation at 95°C to activate the GoTaq enzyme, followed by 45 cycles of denaturation at 95°C for 15 s, annealing at 56°C for 20 s, and extension at 72°C for 25 s, and the fluorescence signal was acquired at the end of every cycle. At the end, a final extension was performed at 72°C for 8 min, following the gene melting analysis. The experiment details were described in our previous study [9].

### Skewed clonal expansion of TCRBV gene families

After the gene melting step was complete, the melting curve for the 24 TCRBV gene families was obtained. The melting peak can be determined by plotting the negative first derivative of the decrease in fluorescence signal versus temperature (-dF/dT) versus temperature (Tm); this technique is called the Gene Melting Spectral Pattern (GMSP) analysis as we previously reported [9]. The skewed clonal expansion was defined using the profile of each GMSP image displayed by the software (Opticon Monitor 3.0) attached to the MJ Opticon 2 DNA engine (Bio-rad, USA), and included two categories: 1) "Oligoclonal expansion", appearing as a main peak associated with other small peaks on the GMSP, and a small peak with a height less than half the height of the main peak; and 2) the "Monoclonal" category, with only one main peak, and a very short small peak or no additional peak.

### Cloning and sequencing

When the GMSP analysis of a sample demonstrated a TCRBV family monoclonal profile (single peak), the corresponding TCRBV gene family was selected for cloning and sequencing to characterize and determine the degree of homogeneity within the CDR3 region. The brief steps were following, the cDNA of the TCRBV gene family was amplified again using GoTaq DNA polymerase (Promega, USA) under the conditions: pre-incubation at 95°C for 2 min to denature target cDNA, 95°C for 40 s, annealing at 56°C for 40 s, and extension at 72°C for 60 s, for 40 cycles of amplification. PCR products were separated using 2% (0.5% Tris-buffered EDTA) agarose gels (FMV BioProducts, Rockland, ME), excised, and purified using a QIAEX gel extraction kit (Qiagen, German). Purified products were ligated into pGEMT-T easy vector using the pGEM-3Z Cloning Kit (Promega, USA) according to the manufacturer's instructions. The plasmid DNA was sequenced in an ABI3730 DNA Sequencer (Applied Biosystems, USA). The results sequenced were analyzed against a standard TCRBV gene database http://www.imgt.org.

### Statistical analysis

Differences in data between two groups were examined using a χ^2^-test or Student's *t*-test, with a *P *< 0.05 considered significant. Statistical analysis was performed using SPSS 16.0 software (SPSS Inc., Chicago, USA).

## Results

### Skewed TCRBV repertoire within total PBMCs and CD8^+^, CD8^- ^subsets

**Figure 1:**
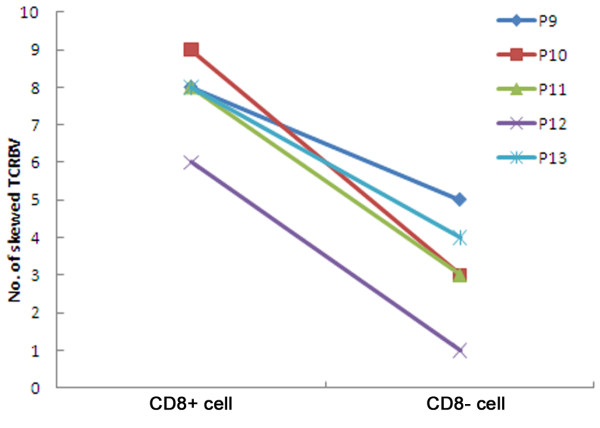
**Comparing the number of skewed TCRBV CDR3 between CD8^+ ^and CD8^- ^cell subsets in representative CHSB patients (corresponding to P9-13, Table 3)**. Data for additional comparisons are provided in table 3. CD8^+ ^cell, purified CD8^+ ^T lymphocytes; CD8^- ^cell, PBMCs depleted of CD8^+ ^cells; CSHB, chronic severe hepatitis B.

The CD8^+ ^and CD8^- ^PBMC, which were mostly CD4^+^, were obtained using magnetic sorting. The TCRBV CDR3 profiles were compared between two sorted cell populations (15 samples sorted) and the 27 PBMCs populations isolated from patients with CSHB. Among the three groups of cell populations evaluated, the TCRBV families in the CD8^+ ^cells from patients contained a greater number of skewed-clonally expanded TCRBV families. The average number of skewed (oligoclonal and monoclonal) TCRBV families in the CD8^- ^PBMC fraction was lower than the PBMCs and the CD8^+ ^subset (*P *< 0.01), and there was no significantly difference between the two latter groups (*P *> 0.05). Additionally, there were a higher number of patients with a normal TCRBV pattern in the CD8^- ^fraction compared to the other two cell fractions (Table 2). There was a greater number of skewed clone expansion of TCRBV families expressed in the CD8^+ ^PBMC compared to the CD8^- ^cells (Table 3, Figure 1).

**Table 2:** The frequency of skewed TCRBV in CD8^+ ^and CD8^- ^cells (PBMCs excluding CD8^+ ^cell) and PBMCs from patients with CSHB^a^.

TCRBV families	**CD8^+ ^cell Incidence (%)**^**b**^	**CD8^- ^PBMC Incidence (%)**^**b**^	**Total PBMCs Incidence (%)**^**b**^
1	2	0	3
2	4 (28.6)	1	4
3	3	0	4
4	3	3	6
5.1	4 (28.6)	3	7 (28)
5.2	5 (35.7)	1	4
6	3	0	4
7	4 (28.6)	5 (38.5)	9 (36)
8	2	0	7 (28)
9	2	0	6
10	0	1	2
11	9 (64.3)	9 (69.2)	15 (60)
12	5 (35.7)	2	6
13.1	3	1	7 (28)
13.2	5 (35.7)	0	7 (28)
14	2	0	3
15	2	1	2
16	3	0	3
17	5 (35.7)	2	5
18	5 (35.7)	1	8 (32)
19	0	0	1
20	4 (28.6)	4 (30.1)	4
21	4 (28.6)	1	3
22	6 (42.9)	3	5
23	1	2	5
24	3	1	1
Total no. of skewed Vβ (average ratio for a case)	89 (6.36)^c, d^	41 (3.15)^c^	131 (5.24)^c, d^
No. of patients examined with normal pattern (ratio, %)	1 (6.67)^e^	2 (13.33)^e^	2 (7.41)^e^
No. of patients examined	15	15	27

^a^The number of TCRBV gene families showing skewed-clone expansion (oligoclonal or monoclonal) is summarized in patients with CSHB.

^b^The number of samples showing a skewed-clone expansion in total detected samples (percentages of each TCRBV skewed-clone expansion). Samples with normal GMSP (no skewed-clone expansion pattern) are excluded in the percentage calculation.

^c^The average expansion rate of TCRBV gene families was lower in CD8^- ^PBMC (depleted CD8^+ ^cells) than in other two cell populations (*P *< 0.01 by *χ^2 ^*test). ^d^There was no significant difference between the two groups (*P *> 0.05 by *χ^2 ^*test). Sample with normal GMSP (no skewed-clone expansion pattern) are excluded in the average ratio calculation.

^e^The incidence of the normal GMSP was significantly higher in CD8^- ^PBMC (depleted CD8^+ ^cells) than in other two cell populations (*P *< 0.01 by *χ^2 ^*test).

**Table 3:** GMSP assay-generated profile of skewed TCRBV gene families in CD8^+ ^and CD8^- ^cells in patients with CSHB.

**Patients no**.	CD8^+ ^cells		CD8^- ^cells	
	
	Skewed TCRBV	Monoclone	Skewed TCRBV	Monoclone
1	3, 5.2, 7, 13.1, 18, 20	7, 13.1	7, 11	7, 11
2	5.1, 5.2, 7, 11, 12, 21, 22, 24	11	None	None
3	3, 5.1, 17, 20, 23	None	2, 7, 11, 20	11, 20
4	4, 5.2, 11, 12, 18, 21, 22	11	7, 11	None
5	2, 13.2, 15, 16	2, 16	4, 13.1	None
6	3, 5.2, 17, 22	17	5.1, 11, 17, 22, 23	11
7	4, 6, 8, 11, 12	11	4, 7, 11	4, 7, 11
8	None	None	None	None
9	6, 8, 11, 13.1, 13.2, 14, 16, 22	11, 13.1	5.1, 11, 17, 22, 23	11
10	9, 11, 13.1, 13.2, 16, 17, 21, 22, 24	11, 13.2	5.1, 12, 20	None
11	2, 5.2, 9, 11, 12, 14, 15,18	5.2, 11, 18	11, 20, 24	11, 24
12	3, 7, 11, 13.1, 20, 21	7, 11, 20	4	4
13	2, 5.1, 6, 13.2, 17, 18, 22, 24	6, 17	10, 11, 15, 22	11
14	1, 4, 7, 11, 17, 18	11, 18	5.2, 7, 11, 18, 20	11, 18
15	2, 5.2, 12, 13.2, 20	None	12, 21	None
Total no. of altered (skewed) TCRBV families (no. of patients)	89 (15)^a^	22 (15)^b^	41 (15)^a^	15 (15)^b^

^a ^The rate of skewed TCRBV families was higher in CD8^+ ^cells than in CD8^- ^cells (*P *< 0.01 by *χ^2 ^*test). ^b ^The monoclonal rate was not significantly different between the two groups (*P >*0.05 by *χ^2 ^*test).

### GMSP profile of monoclonal TCRBV in total PBMCs and CD8^+^, CD8^- ^subsets

**Figure 2:**
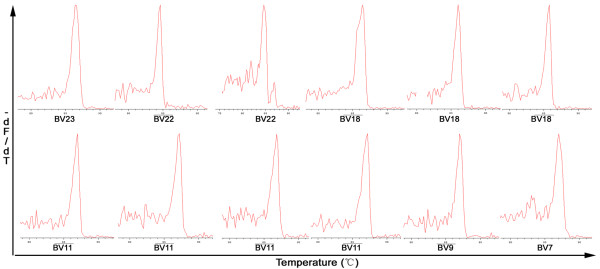
**Representative GMSP with a single-peak (monoclonal expansion) of TCRBV in the PBMCs from patients with CSHB**. The TCRBV gene families shown above in the top graphs correspond to P69 (BV23), P90 (BV22), P75 (BV22), P90 (BV18), P75 (BV18), and P105 (BV18); the TCRBVs shown on the bottom graphs correspond to P107 (BV11), P131 (BV11), P83 (BV11), P71 (BV11), P69 (BV9) and P172 (BV7). The corresponding amino acid sequences are shown in Table 4. The melting temperature is on the x-axis of each plot. The negative first derivation of the decrease in fluorescence versus temperature (-dF/dT) is shown on the y-axis.

**Figure 3:**
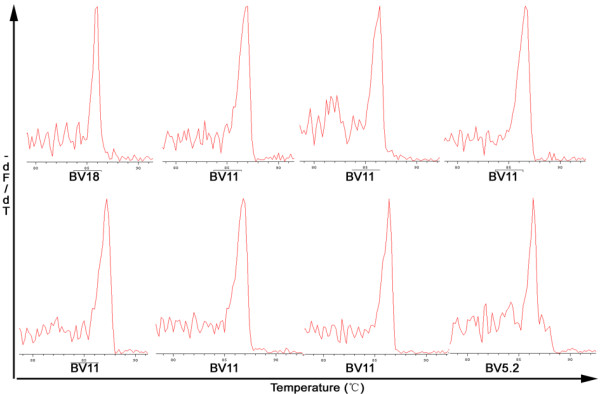
**Representative GMSP with single-peak (monoclonal expansion) TCRBV in the CD8^+ ^cell and CD8^- ^cell from patients with CSHB**. The TCRBV gene families shown on the top graphs correspond to P89CD8^+ ^(BV18), P133CD8^+ ^(BV11), P115CD8^+ ^(BV11), and P84CD8^+ ^(BV1); the TCRBVs shown in the bottom graphs correspond to P119CD8^- ^(BV11), P89CD8^- ^(BV11), P84CD8^- ^(BV11), and P89CD8^+ ^(BV5.2). The corresponding amino acid sequences are shown in Table 5. The melting temperature is on the x-axis of each plot. The negative first derivation of the decrease in fluorescence versus temperature (-dF/dT) is shown on the y-axis.

The profile of the TCRBV families expressed by total PBMCs and CD8^+^, CD8^- ^fractions was determined using GMSP analysis. Although, the number of single-peaks and biased-peaks for the 24 TCRBV families detected from the three cell populations (total PBMCs and CD8^+^, CD8^- ^cell subsets) differed among the CSHB patients, the two skewed TCRBV gene families (BV7, BV11) were more prevalent than other TCRBV families (Table 2 and 3). A representative GMSP profile of a monoclonal TCRBV expressed in PBMCs and in the CD8^+ ^and CD8^- ^subsets is shown in Figures 2 and 3, respectively.

### TCRBV CDR3 amino acid motifs in total PBMCs and CD8^+^, CD8^- ^subsets

The monoclonal expansion of TCRBV CDR3 gene families were cloned, sequenced, and translated to corresponding amino acid sequences. The representative amino acid sequences of the TCRBV CDR3 from PBMCs and CD8^+ ^and CD8^- ^subsets from patients with CSHB are shown in Table 4 and 5. We found that, among different CSHB patients, the monoclonal expansion of TCRBV CDR3, such as BV22 (DLGVAQ with BJ2.7), BV18 (RTGDTEA with BJ1.1) and BV11 (AGEL with BJ2.2 or VYNEQ with BJ2.1), in PBMCs was similar. However, when we compared the monoclonal expansion of TCRBV CDR3 in the CD8^+ ^and CD8^- ^cell subsets, the amino acid sequence of TCRBV18 CDR3, (RTGDTEA with BJ1.1), was similar to PBMCs, as was the sequences of TCRBV11 (AGEL with BJ2.2 or VYNEQ with BJ2.1) and TCRBV5.2 (LTAGAYTGEL with BJ2.2).

**Table 4:** Representative amino acid sequences of monoclonal TCRBV families in PBMCs from CSHB patients.

Patients	Vbeta		CDR3	BJ		Ratio
69	BV23	SALYFCASS	VEGGNTI	YFGEGSWLTVVED	1.3	21/21
90	BV22	SAMYFCASS	DLGVAQ	YFGPGTRLTVTED	2.7	9/20
75	BV22	SAMYFCASS	DLGVAQ	YFGPGTRLTVTED	2.7	4/18
90	BV18	SAAYFCASS	RTGDTEA	FFGQGTRLTVVED	1.1	21/21
75	BV18	SAAYFCASS	RTGDTEA	FFGQGTRLTVVED	1.1	21/21
105	BV18	SAAYFCASS	RTGDTEA	FFGQGTRLTVVED	1.1	21/21
107	BV11	SQYLCASS	AGEL	FFGEGSRLTVLED	2.2	5/21
131	BV11	SQYLCASS	AGEL	FFGEGSRLTVLED	2.2	9/20
138	BV11	SQYLCASS	AGEL	FFGEGSRLTVLE	2.2	9/18
172	BV11	SQYLCASS	AGEL	FFGEGSRLTVLED	2.2	3/21
172	BV11	SQYLCATG	VYNEQ	FFGPGTRLTVLED	2.1	4/21
75	BV11	SQYLCATG	VYNEQ	FFGPGTRLTVLED	2.1	7/8
83	BV11	SQYLCATG	VYNEQ	FFGPGTRLTVLED	2.1	20/20
71	BV11	SQYLCATG	VYNEQ	FFGPGTRLTVLED	2.1	21/21
69	BV9	SAVYFCASS	LQAGRGEQ	FFGPGTRLTVLED	2.1	12/20
172	BV7	SALYLCASS	QDSVTTGAQ	YFGPGTRLLVLED	2.5	15/21
90	BV6	SAVYLCASS	LAWEEQETQ	YFGPGTRLLVLED	2.5	21/21
81	BV5.2	SALYLCASS	LTAGAYTGEL	FFGEGSRLTVLED	2.2	10/22
2	BV5.1	SALYLCASS	LEWGASYEQ	YFGPGTRLTVTED	2.7	9/20

When the GMSP displayed a single peak (Figure 2), the cDNA of corresponding TCRBV family was re-amplified and PCR product was sequenced after cloning. The amino acid sequences of the max rate of TCRBV families are shown.

**Table 5:** Representative amino acid sequences of monoclonal TCRBV families in CD8^+ ^and CD8^- ^cells (depleted CD8^+ ^cells) from CHSB patients.

**Patients**^**a**^	Vbeta		CDR3	BJ		Ratio
89 (CD8^+^)	BV18	SAAYFCVSS	RTGDTEA	FFGQGTRLTVVED	1.1	11/11
133 (CD8^+^)	BV11	SQYLCASS	AGEL	FFGEGSRLTVLED	2.2	11/20
115 (CD8^+^)	BV11	SQYLCATG	VYNEQ	FFGPGTRLTVLED	2.1	14/16
84 (CD8^+^)	BV11	SQYLCATG	VYNEQ	FFGPGTRLTVLED	2.1	27/27
119 (CD8^-^)	BV11	SQYLCASS	AGEL	FFGEGSRLTVLED	2.2	14/22
89 (CD8^-^)	BV11	SQYLCATG	VYNEQ	FFGPGTRLTVLED	2.1	18/20
84 (CD8^-^)	BV11	SQYLCATG	VYNEQ	FFGPGTRLTVLED	2.1	17/20
89 (CD8^+^)	BV5.2	SALYLCASS	LTAGAYTGEL	FFGEGSRLTVLED	2.2	11/29

When the GMSP displayed a single peak (Figure 3), the cDNA of corresponding TCRBV family was re-amplified and PCR product was sequenced after cloning. The amino acid sequences of the max rate of TCRBV families are shown. ^a ^The cell subset for analysis in brackets.

## Discussion

HBV infects a narrow host range, and the lack of ideal animal models or the efficient cell culture system greatly limits the study of the HBV-specific immune response [10]. In recent years, research results show that the HBV antigen-induced cellular immune response necessary for viral clearance, especially the activity of HBV-specific CTL, also mediates HBV-associated liver inflammation and damage [11,12].

T lymphocytes are composed of mainly CD4^+ ^and CD8^+ ^cells. There are characteristic TCR molecular chains expressed on the surface of T cells. In peripheral blood from healthy donors, the TCR of more than 95% of T cells is composed of alpha and beta chain heterodimers, i.e., αβT cells [13]. Each T cell clone expresses a unique TCR that recognizes antigen-derived peptide bound to major histocompatibility complex (MHC), leading to T cell activation, proliferation, and effector function. The TCR has three complementary determining regions (CDR1, CDR2 and CDR3). CDR3 is the key determinant of T cell antigen specificity and mediates T cell diversity [4,14]. Therefore, analysis of the CDR3 profile reflects changes in the T cell population stimulated by a specific antigen [15,16]. This may aid in determining the effectiveness of the T cell response. For analysis of the TCR repertoire, the β chain is often preferred because of the lower number of gene families associated even though a higher overall sequence variability of sequence compared to the α-chain has been reported [17].

Several approaches have been used to determine the expansion of TCRBV subgroups including Northern blotting, semi-quantitative PCR using radioisotope-conjugated probes [18], or fluorescence activated cell sorting (FACS) using fluorochrome-conjugated monoclonal antibodies (mAbs) specific to TCRBV subgroups [19] or direct quantification and characterization of CD8^+ ^T cells using tetramer staining and intracellular cytokine staining. At present, the spectratyping (immunoscope) based on PCR was developed as the current standard in TCRBV repertoire analysis [4,16]. However, these approaches are susceptible to cross-contamination, laborious, and time consuming because they require PCR amplification, isolation of individual bands based on DNA size, and purification, followed by repeated cloning and sequencing.

Recently, quantitative real-time reverse transcription PCR (qRT-PCR) has become a widely accepted method for rapid and reproducible quantification of gene expression. Most previous attempts to quantify TCRBV expression using qRT-PCR have utilized one primer directed at the gene encoding the TCR constant region of the beta chain (BC) and another primer or fluorogenic probe directed towards the gene encoding the beta chain variable region (BV) [20-22]. In the current study, we examined the molecular features of the CDR3 motif from isolated PBMCs and CD8^+ ^and CD8^- ^subsets from patients with CSHB using GMSP analysis. The GMSP methods were based on qRT-PCR with the gene melting curve technique and developed for detecting the TCRBV gene family as reported in our previous publication [9].

In peripheral blood, the number of CD4^+ ^T cells is approximated 1.5 times greater than the number of the CD8^+ ^T cell; this may impact analysis of TCR repertoire. In this study, we directly analyzed the degree of skewed clonally expanded TCRBV families in purified PBMCs from 27 CSHB patients. We found that the average number of skewed TCRBV families was 5.24, but within the CD8^+ ^and CD8^- ^subsets, the average number was 6.36 and 3.15, respectively. In summary, the number of skewed TCRBV in the CD8^- ^fraction was significantly lower than in the CD8^+ ^cells. This is consistent with previous reports demonstrating that proliferating HBV-antigen specific T cells were CD8^+ ^T cells rather than CD4^+ ^[23]. The skewed TCRBV families among the three cell groups were different, implying that it may be more accurate to analyze the molecular features of the CDR3 motif in sorted T cells. Although the positive selection of CD8^+ ^cells results in a CD8^- ^fraction that contains a residual number of CD8^+ ^cells and includes B cells, natural killer cells and some mononuclear cells, this "contamination" has relatively little impact on the GMSP profile of the TCRBV CDR3s within the total CD8^- ^cell subset [19].

In recent years, TCR gene transfer has been developed as a reliable method to generate large numbers of T cells ex vivo with given antigen-specificity and functional avidity, providing great prospects for clinical application [24-29]. TCR re-directed HBV-specific T cells generated from the PBMCs of chronic HBV and HBV-related HCC patients were shown to be multifunctional and capable of recognizing HBV infected cells and HCC tumor cells expressing viral antigens from naturally integrated HBV DNA [30]. Thus, characterization of the HBV-specific T cells, especially their TCR gene usage [31], is essential for the elucidation of the pathogenesis of HBV infection and for the development of individualized treatment.

In the current study, we found that the monoclonal populations expressing TCRBV7 or BV11 molecule were more prevalent compared with other TCRBV families used, regardless of the CDR3 in PBMCs, CD8^+ ^or CD8^- ^subsets from CSHB patients. We also found the two CDR3 amino acid sequences had conserved motif (BV11, AGEL or VYNEQ and BV7, QDSVTTGAQ), and, among different CSHB patients, the amino acid motifs in the TCRBV22, BV18 and BV11 CDR3 in PBMCs were similar, respectively. At present, although, it is not clear if or how the emergence of the TCRBV families influences the course of CSHB, in a follow-up study, we observed an interesting phenomenon, when CSHB patients expressed the TCRBV11 CDR3 amino acid sequence "AGEL", their short-term response to treatment was better than patients expressing the TCRBV11 CDR3 sequence "VYNEQ". Moreover, the relatively conservative TCRBV gene families may help produce antigen-specific T cells using TCR gene transfer for the treatment of liver disease.

## Conclusion

In this report, the molecular features of the CDR3 motif in PBMCs and in CD8^+ ^and CD8^- ^subsets isolated from patients with CSHB were explored using GMSP analyses. During inflammatory episodes in patients with HBV infection, the skewed TCRBV in CD8^+ ^subset was significantly larger than in the CD8^- ^subset. This may help to explain the underlying mechanisms of HBV pathogenesis and may be related to impaired viral clearance and repeated HBV infection [32], although, this requires further investigation. The identification of the relatively conserved amino acid sequences of the TCRBV7, BV11 and other member of TCRBV family expressed in this patient population could be used in evaluating the health status of patients with CSHB, and may aid in the development of transduction gene therapy for patients infected with HBV.

## Abbreviations

ALT: alanine amino transferase; AST: aspartate amino transferase; HBV: hepatitis B virus; HBsAg: hepatitis B surface antigen; HBeAg: hepatitis B envelope antigen; CSHB: chronic severe hepatitis B; Real-time-PCR: real-time fluorescent quantitative polymerase chain reaction; GMSP: gene melting spectral pattern.

## Competing interests

The authors declare that they have no competing interests.

## Authors' contributions

JZ conducted the study, participated in the data collection, performed most experiments, and wrote the initial draft and revised the manuscripts. JQ and HF collected the preliminary data, and helped to perform some experiments. WL and SJ participated in the study design and interpretation of the data. BH and HY assisted in experimental design and help to interpret data. LJ study coordination and revision of the paper. All authors read and approved the final manuscript.
